# Excess Enthalpies for Binary Mixtures of the Reactive System Acetic Acid + n-Butanol + n-Butyl Acetate + Water: Brief Data Review and Results at 313.15 K and Atmospheric Pressure

**DOI:** 10.3390/ijms24065137

**Published:** 2023-03-07

**Authors:** Alexandra Golikova, Anna Shasherina, Yuri Anufrikov, Georgii Misikov, Maria Toikka, Irina Zvereva, Alexander Toikka

**Affiliations:** Institute of Chemistry, St. Petersburg State University, Universitetskiy Prospect 26, Peterhof, Saint Petersburg 198504, Russia

**Keywords:** heat of mixing, calorimetry, n-butyl acetate, thermochemistry, Redlich–Kister equation, NRTL

## Abstract

The data on molar excess enthalpies, $H_{m}^{E}$, for the binary mixtures acetic acid + n-butanol, acetic acid + n-butyl acetate and n-butanol + n-butyl acetate at 313.15 K and atmospheric pressure were obtained with use of the C80 isothermal mixing calorimeter (Setaram). The correlation of the data was carried out using the NRTL model and Redlich–Kister equation. A comparative analysis with the literature data on all available binary subsystems of the quaternary system was carried out. Other thermodynamic properties ($C_{p,m}^{E}$, $S_{m}^{E}$, $\Delta_{mix}S_{m}$, $G_{m}^{E}$ and $\Delta_{mix}G_{m}$) of the binary systems were estimated using literature data and well-known formulas of classical thermodynamics.

## 1. Introduction

Features of molecular interaction in a solution can be described using thermodynamic characteristics, which primarily include the molar excess enthalpy. The excess enthalpies are the direct information about the energetic effects occurring between the molecules present in the mixtures. Such research is necessary not only for improving the solution theories and the development of appropriate databases, but also for describing the nature of the processes.

The data sets on enthalpy include important thermodynamic properties in many engineering applications and for the development of chemical engineering processes. Thus, excess thermodynamic properties in general quantify the deviations from ideality of the thermodynamic functions of mixtures.

Esters are promising substances for such studies. These substances are associated with a broad market in the chemical industry. One of the most common solvents of the paint and coatings industry, as well as a dehydrant in a number of industrial applications [1,2], is n-butyl acetate. In addition, n-butyl acetate is perfectly suitable as a solvent for the environment [3]. It is used in fragrances in cosmetic product, pharmaceutical and food industries [2]. One of the important properties of n-butyl acetate is its ability to act as an additive to gasoline and diesel fuel, and as biofuel [4,5].

Due to the possibility of using n-butyl acetate in various industries, systems with n-butyl acetate are among the most studied. Quite common are solubility, chemical equilibrium, vapor–liquid and liquid–liquid equilibrium and critical states studies for n-butyl acetate system synthesis [6,7,8,9,10] at deferent temperatures, e.g., 308.15, 318.15, 328.15 K, mostly at atmospheric pressure (excluding vapor–liquid experiments). In our work, we decided to pay attention to the thermal characteristics of this system. Despite the seemingly relatively large amount of data on excess enthalpies for the system acetic acid + n-butanol + n-butyl acetate + water, after a careful review of the literature, it turned out that the available results are not enough for a complete thermodynamic picture of the behavior of the system as a whole. For the convenience of perceiving the data on molar excess enthalpy available in the literature for the quaternary system investigated, including binary subsystems, we have collected them in Table 1.

For the acetic acid + n-butanol system, experimental data in the temperature range 298.15–318.15 are presented by the authors of the articles [11,12,13]. Authors [12] have published the results as the function of excess enthalpies (*H^E^*) on temperature and composition, and they fit the experimental data to the power series. In [13], the NRTL parameters were calculated for all experimental results, and it should be noted that temperatures indicated in the text differ from the ones in tables. Experimental data on excess enthalpies for the system acetic acid + n-butyl acetate were published only by the authors of [13]. There is a sufficient amount of data in the literature concerning the system acetic acid + water. Experimental results are presented in [17]. The authors of [14,18,19] fitted experimental data with different equations. The corresponding graphs are plotted in [14] for all temperatures except 298.15 K (the latter is listed in the table), but results for this temperature can be found in [32]. Data on excess enthalpy and computed the molar excess entropy values are listed in [20]. In [15,16,21], the data were obtained using methods to predict the molar excess enthalpies. In [13,22,23,24,25], the information on experimental molar excess enthalpy for the n-butanol + n-butyl acetate system was collected and results have also been correlated using various equations and models. The authors of [26] calculated the molar excess enthalpies and presented them in diagrams. The experimental molar excess enthalpies of mixing for the system n-butanol + water are represented with dots in graphs only in [27]. These data were also obtained by the authors of [28,29,30]. There is a fairly limited amount of data on molar excess enthalpy for the n-butyl acetate + water system in the literature. Experimental and calculated results are presented by the authors of [13,31]. Data on molar excess enthalpy for the acetic acid + n-butanol + n-butyl acetate + water system are presented in [13]. There are no data on the excess enthalpies for all ternary subsystems in the literature. 

As a result of this study, we provide new experimental data on the excess enthalpies of mixing for binary subsystems of the acetic acid + n-butanol + n-butyl acetate + water system. Obtained data were correlated using the local composition NRTL model that is well known to be a good approach for correlating experimentally measured thermodynamic properties of various systems with different natures of deviation from the ideal solution. Due to the fact the NRTL model is a thermodynamically consistent model of local composition, it is widely used not only for the case of thermodynamic analysis of organic mixtures, but also within the chemical engineering field. We conducted a broad comparative analysis of the results available in the literature on this topic, as a result of which it was found that the data have a strong discrepancy among themselves. Additionally, thermodynamic functions, such as molar excess entropy, molar excess heat capacity, molar excess Gibbs energy, molar entropy of mixing and molar Gibbs energy of mixing, were also evaluated.

## 2. Results and Discussion

**Figure 1:**
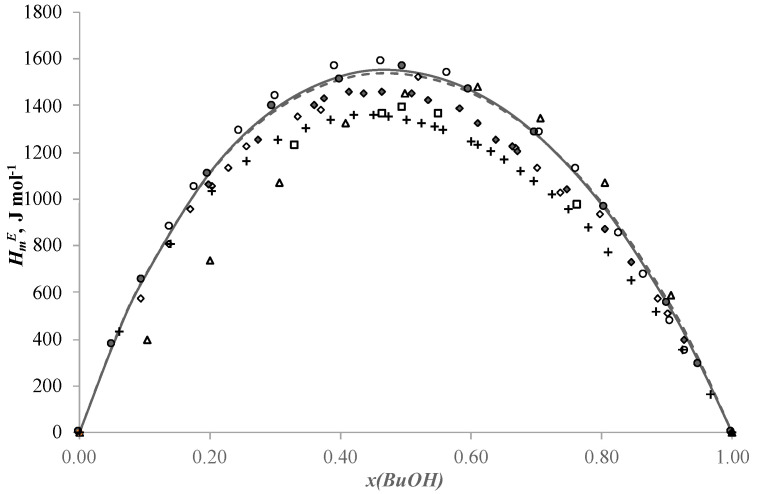
Molar excess enthalpies for binary system n-butanol + n-butyl acetate, (J mol^−1^): the experimental solid circles (●) at 313.15 K, redrawn from Ref. [23] open up triangles (△) at 298.15 K, redrawn from Ref. [24] open rhomb (◊) at 298.15 K, redrawn from Ref. [22] solid rhomb (⬥) at 298.15 K, redrawn from Ref. [25] plus (+) at 303.15 K, redrawn from Ref. [24] open rectangle (□) at 313.15 K, Ref. [13] open circles (○) at 313.15 K, calculated by Redlich-Kister equation (―) and NRTL model (---), *x*—mole fraction of n-butanol.

**Figure 2:**
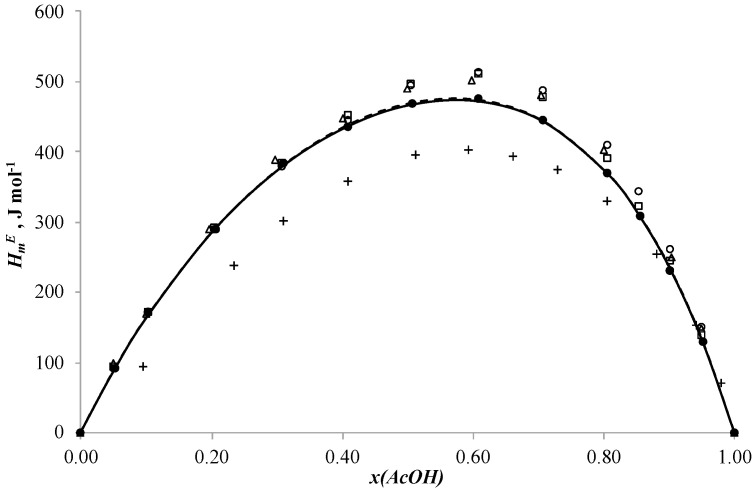
Molar excess enthalpies for binary system acetic acid + n-butanol, (J mol^−1^): the experimental solid circles (●) at 313.15 K, redrawn from Ref. [12] open circles (○) at 298.15 K, redrawn from Ref. [11] plus (+) at 298.15 K, redrawn from Ref. [13] open up triangles (△) at 313.15 K, redrawn from Ref. [12] open rectangle (□) at 318.15 K, calculated by Redlich-Kister equation (―) and NRTL model (---), *x*—mole fraction of acetic acid.

**Figure 3:**
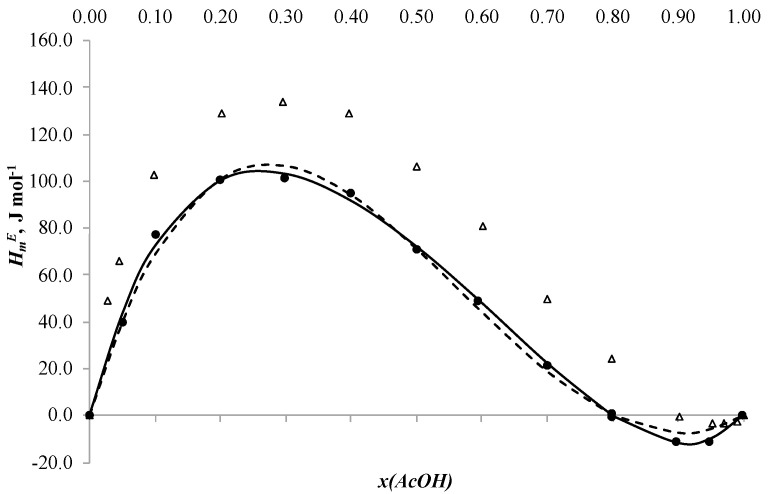
Molar excess enthalpies for binary system acetic acid + n-butyl acetate, (J mol^−1^): experimental solid circles (●) at 313.15 K, redrawn from Ref. [13], open triangles (△) at 313.15 K, calculated by Redlich–Kister equation (―) and NRTL model (---), *x*—mole fraction of acetic acid.

The new results for the binary systems acetic acid + n-butanol, acetic acid + n-butyl acetate and n-butanol + n-butyl acetate are given in Table 2, Table 3 and Table 4, respectively, and plotted in Figure 1, Figure 2 and Figure 3.

The molar excess enthalpies for binary systems n-butanol + n-butyl acetate and acetic acid + n-butanol (Figure 1 and Figure 2) are positive in all ranges of mole fraction. The maximums are $H_{m}^{E}$ = 1556.3 J mol^−1^ at $x_{BuOH}$ = 0.4967 in the n-butanol + n-butyl acetate system and $H_{m}^{E}$ = 475.2 J mol^−1^ at $x_{AcOH}$ = 0.6092 in the acetic acid + n-butanol system. The curves change almost symmetrically. This shape of the curves can be explained by the fact that at the beginning of the mixing processes the hydrogen bonds are stronger than at the end of the processes [33]. The dependence of the molar excess enthalpy on composition for the system acetic acid + butyl acetate (Figure 3) has an S-shape with small exothermic effect on the side of acetic acid. The minimum is $H_{m}^{E}$ = −11.2 J mol^−1^ at $x_{AcOH}$ = 0.9496 and the maximum is $H_{m}^{E}$ = 100.7 J mol^−1^ at $x_{BuOH}$ = 0.2994. Such a change in the shape of the curve of dependence of molar excess enthalpy on composition is explained by the breakage of hydrogen bonds in acetic acid and their formation in the final mixture between acid and ether [33,34].

As can be seen from Figure 1, the data for the n-butanol + n-butyl acetate system have some discrepancy. Figure 1 shows that the results at 298.15 K given in [22] (solid rhomb (♦)) coincide with the data obtained in [24] (open rhomb (◊)) at the same temperature only at low and high concentrations of substances. The points from [22] have lower values of heats in the area of equal component ratios. The results reported by the authors in [23] (open triangle (△)) are shifted to the right according to the schedule. The results presented in [25] (plus (+)) at 303.15K are lower than all the presented data obtained at 298.15 K, and these data lie close to the results provided by the authors of [24] (open rectangle (□)) at 313.15 K in the area of high n-butanol concentrations. The thermogram taken from [24] at 313.15 K lies much lower than all other thermograms obtained at this temperature. The data obtained in this work (solid circle (●)) are in good agreement with the data results from [13] (open circle (o)).

The dependence of molar excess enthalpy on the concentration of n-butanol for the acetic acid + n-butanol system is shown in Figure 2. The data obtained for this system have a strong discrepancy among themselves. The values of molar excess enthalpy obtained in [11] (plus (+)) at 298.15 K are much lower than the data obtained in [12] (open circle (○)) at this temperature. The thermograms plotted in [12] (open rectangle (□)) at 318.15 K lie very close to the data obtained in [13] (open triangle (△)) at 313.15 K. The results given in our work (solid circle (●)) at 313.15 K lie below the data obtained in [13] at the same temperature, which indicates data inconsistency.

## 3. Materials and Methods

### 3.1. Materials

The purities of acetic acid, n-butanol, n-butyl acetate and water used in the study were determined by a gas chromatography (GC) method with a Shimadzu GC-2010 plus including comparison of the measured refractive indexes, $n_{D}$, with the literature values [35]. The refractive indexes were determined with the IRF-454B2M refractometer (“KOMZ”, Russia). The chemical specifications are summarized in Table 5.

### 3.2. Molar Excess Enthalpy Measurements

The study of excess enthalpy of mixing in binary subsystems of the quaternary system acetic acid + n-butanol + n-butyl acetate + water was performed with use of the C80 isothermal mixing calorimeter (Setaram). The measurements were carried out at the temperature 313.15 ± 0.05 K with concentration step of 0.1 in the scale of mole fraction. A membrane mixing cell (material—stainless steel) was used for experimental measurements. At the beginning of the experiment, two pure components were separated from each other by a membrane. Upon achievement of calorimeter signal stabilization, the membrane was destroyed by special rod inside the cell, and the resulting heat flow was precisely measured by a Calvet sensor. The calculation of the heat effect of mixing was fulfilled with the use of the coefficient obtained from Joule effect calibration (electrical calibration). The standard system hexane + cyclohexane was used for testing of the apparatus and procedure. The experimental process was described in more detail in our previous works [34,36]. The relative uncertainty *U_r_* for excess enthalpies is *U_r_*($H_{m}^{E}$) = 0.03.

The work Investigates the heats of mixing of the acetic acid + n-butanol reaction system. The esterification reaction proceeds very slowly in the absence of a strong acid as a catalyst, this is discussed in [11,36,37]. In addition, experiments were carried out in [34] for the system n-propanol + acetic acid, which has proven to have an extremely slow chemical reaction in the absence of a catalyst. In this regard, the effect of the reaction enthalpy on calorimetric measurements can be neglected.

### 3.3. Calculation

#### 3.3.1. Redlich–Kister

Obtained experimental data were correlated with use of the Redlich–Kister equation in order to check their values for thermodynamic correspondence [38]:

(1) $$H_{m,ij}^{E}=x_{i}x_{j}\sum_{k=0}^{N}A_{k}{\left(x_{i}-x_{j}\right)}^{k}$$

where *x_i_*, *x_j_*—mole fraction of component *i* and *j*, *A_k_*—the adjustable parameters, *N*—the polynomial degree. The classical formula of the Redlich–Kister equation is used to correlate the symmetric dependencies for the systems acetic acid + n-butanol, acetic acid + n-butyl acetate and n-butanol + n-butyl acetate. The simulation results are shown in Figure 1, Figure 2 and Figure 3, respectively. 

To characterize the best description of the Redlich–Kister equation by a polynomial for a set of experimental points, the standard deviation parameter was used:

(2) $$\sigma \left(H^{E}\right)=\sqrt{\frac{\sum_{i=1}^{n}{\left(H_{calc,i}^{E}-H_{exp,i}^{E}\right)}^{2}}{n-N}}$$

where *n* is number of experimental points, *N* is number of coefficients of the polynomial. The average calculation error was estimated using the formula

(3) $$ARD\left(\%\right)=\frac{100}{n}\sum_{i=1}^{n}\frac{\left|H_{calc,i}^{E}-H_{exp,i}^{E}\right|}{\left|H_{exp,i}^{E}\right|}$$

Parameters of these equations, average relative deviation (*ARD*) and standard deviation (*σ*(*H^E^)*) are presented in Table 6.

#### 3.3.2. NRTL

The NRTL model [39] was used to approximate experimental results on the enthalpies of mixing binary systems:

(4) $$H^{E}=x_{1}x_{2}\left[\frac{G_{21}\Delta g_{21}\left(x_{1}+x_{2}G_{21}-x_{1}\tau_{21}\alpha_{12}\right)}{{\left(x_{1}+x_{2}G_{21}\right)}^{2}}+\frac{G_{12}\Delta g_{12}\left(x_{2}+x_{1}G_{12}-x_{2}\tau_{12}\alpha_{12}\right)}{{\left(x_{1}+x_{2}G_{21}\right)}^{2}}\right],$$

where

(5) $$G_{12}=exp\left(-\alpha_{12}\tau_{12}\right),\;G_{21}=exp\left(-\alpha_{12}\tau_{21}\right),\;\tau_{12}=\frac{\Delta g_{12}}{RT},\;\tau_{21}=\frac{\Delta g_{21}}{RT},\;G_{12}=exp\frac{\Delta g_{12}}{RT},$$

where $\Delta g_{12}=g_{12}-g_{22}$ and $\Delta g_{21}=g_{21}-g_{11}$ are adjustable binary parameters, and *α*_12_ is the non-randomness parameter.

When finding the coefficients of the equation, the objective function, *OF*, was minimized:

(6) $$OF=\overset{n}{\underset{i=1}{\sum }}{\left(\frac{H_{calc,\;i}^{E}-H_{exp,\;i}^{E}}{H_{exp,\;i}^{E}}\right)}^{2}$$

where the summation is over all *i* data points.

Parameters of the NRTL model and *ARD* values are given in the Table 7 and plotted in Figure 1, Figure 2 and Figure 3.

#### 3.3.3. Estimation of Thermodynamic Functions

For the system acetic acid + n-butanol, we made an attempt to perform estimation of other thermodynamic properties using literature data for *H^E^* at different temperatures. It is a matter of common observation that all the correlations between enthalpy and other thermodynamic functions are true for the molar excess functions and molar functions of mixing as well, because of the fact that molar excess functions are the differences between values of a thermodynamic property of a real system and an ideal one:

(7) $$H_{m}^{E}=H_{m}^{real}-H_{m}^{id}$$

At the same time, the functions of mixing are the differences between a function of a real system and a corresponding linear combination of functions of pure components:

(8) $$\Delta_{mix}H_{m}=H_{m}^{real}-x_{1}H_{m,1}^{*}-x_{2}H_{m,2}^{*}$$

It is known that the excess enthalpy and molar enthalpy of mixing are equal.

(9) $$\Delta_{mix}H_{m}=H_{m}^{E}$$

That is why, if there are $H_{m}^{E}$ data for different temperatures, some other thermodynamic properties for systems can be estimated. For example, molar excess heat capacity is defined as following derivation:

(10) $$C_{p,m}^{E}={\left(\frac{\partial H_{m}^{E}}{\partial T}\right)}_{p,x}$$

Then, it can be shown with Legendre transformation that:

(11) $$C_{p,m}^{E}={\left(\frac{\partial H_{m}^{E}}{\partial T}\right)}_{p,x}={\left(\frac{\partial \left(G_{m}^{E}+TS_{m}^{E}\right)}{\partial T}\right)}_{p,x}={\left(\frac{\partial G_{m}^{E}}{\partial T}\right)}_{p,x}+S_{m}^{E}{\left(\frac{\partial T}{\partial T}\right)}_{p,x}+T{\left(\frac{\partial S_{m}^{E}}{\partial T}\right)}_{p,x}$$

Excess entropy is a derivation of molar excess Gibbs energy:

(12) $${\left(\frac{\partial G_{m}^{E}}{\partial T}\right)}_{p,x}=-S_{m}^{E}$$

Combining (11) and (12), we have:

(13) $$C_{p,m}^{E}=T{\left(\frac{\partial S_{m}^{E}}{\partial T}\right)}_{p,x}$$

Then, at *p*,*x* = const we have the following equation for the molar excess entropy:

(14) $$dS_{m}^{E}=\frac{C_{p,m}^{E}}{T}dT$$

After integration of (14), we have:

(15) $$S_{T_{2},m}^{E}-S_{T_{1},m}^{E}=\int_{T_{1}}^{T_{2}}\frac{C_{p,m}^{E}}{T}dT$$

For the estimation of the excess entropy with Equation (15), the additional data on $S_{m}^{E}$ at one temperature are necessary. They can be calculated from the fundamental equation for the molar excess Gibbs energy:

(16) $$G_{m}^{E}=H_{m}^{E}-TS_{m}^{E}$$

(17) $$S_{m}^{E}=\frac{H_{m}^{E}-G_{m}^{E}}{T}$$

$H_{m}^{E}$ is experimentally measurable and $G_{m}^{E}$ can be calculated from the vapor–liquid equilibrium data.

For the estimation of the molar excess heat capacity, we used our data at 313.15 K and the literature ones. Data for 298.15 were taken from [11] and for 318.15 from [12]. It was observed that, for the concentration range of acetic acid mole fraction between 0.1 and 0.9, the $H_{m}^{E}$ temperature dependence tends to be linear. As the $H_{m}^{E}-x$ curves have extreme points and values of the $H_{m}^{E}$ for the high and low mole fractions of acid are rather low, the influence of the experimental error becomes rather significant if the mole fraction of any component is extremely high. This can be seen in Figure 2 as well. Therefore, we assumed that the dependence of the excess enthalpy remains linear in those regions as well. The $H_{m}^{E}$ − T lines were plotted by processing literature $H_{m}^{E}$ data with the least square method for every composition. Using linear approximation, we have the following equation for the $H_{m}^{E}$:

(18) $$H_{m}^{E}=kT+b$$

According to (10), the molar excess heat capacity for every composition can be calculated as a slope of the $H_{m}^{E}-T$ line:

(19) $$C_{p,m}^{E}=k$$

The uncertainty of the estimated $C_{p,m}^{E}$ value for each composition was determined as the uncertainty of the slope calculated with least square method:

(20) $$\Delta k=\sqrt{\frac{1}{N-2}\left(\frac{S_{H}^{2}}{S_{T}^{2}}-k^{2}\right)}$$

where number of experimental points *N* = 3, $S_{H}^{2}=\langle H_{m}^{E}{}^{2}\rangle -\langle H_{m}^{E}\rangle {}^{2}$,$\;S_{T}^{2}=\langle T^{2}\rangle -\langle T\rangle^{2}$, <…> denote values averaged over experimental ones. The uncertainties of $C_{p,m}^{E}$ were estimated for every experimental composition (12 compositions) and then averaged over the composition:

(21) $$\Delta C_{p,m}^{E}=\frac{\sum_{i=1}^{n}\Delta k_{i}}{n}$$

where number of experimental compositions *n* = 12. The uncertainty of the estimated $C_{p,m}^{E}$ was 0.5 JK^−1^ mol^−1^. Due to the quite low value of $C_{p,m}^{E}$ itself (average value is 3.4 JK^−1^ mol^−1^), the relative standard uncertainty is quite high (15%), however, because of the fact that literature data on molar excess enthalpy are rather limited, such a result tends to be reasonable.

**Figure 4:**
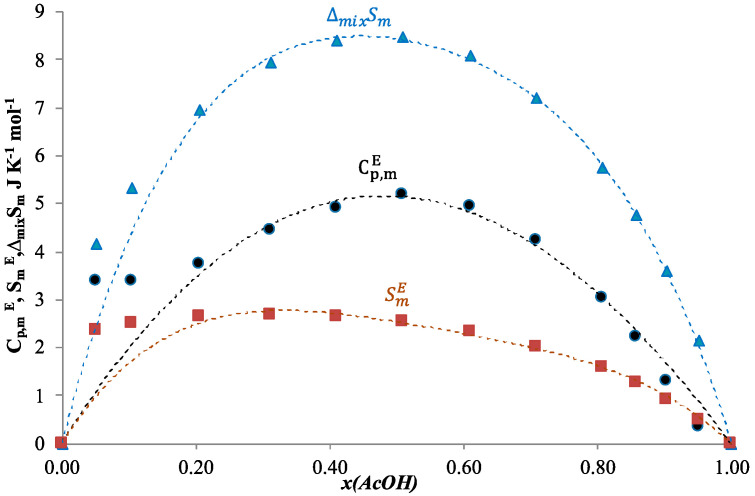
Calculated molar excess heat capacity $C_{p,m}^{E}$, JK^−1^ mol^−1^ (●), molar excess entropy $S_{m}^{E}$, JK^−1^ mol^−1^ (■) and molar entropy of mixing $\Delta_{mix}S_{m}$, JK^−1^ mol^−1^ (▲) for the system acetic acid + n-butanol at 313.15K. The dotted lines (- - -) are the approximation polynomials, *x*—mole fraction of acetic acid.

The calculated $C_{p,m}^{E}$ values at 313.15 K are listed in Table 8 and shown in Figure 4. The dotted lines were obtained with polynomial approximation of the corresponding calculated values. The values of $C_{p,m}^{E}$ are given with accuracy of one tenth according to the estimated uncertainty. According to (9) and (10), it can be shown that $C_{p,m}^{E}$ and $\Delta_{mix}C_{p,m}$ are equal, therefore the curve for $\Delta_{mix}C_{p,m}$ should be the same. Molar excess functions and molar functions of mixing must tend to zero at the points corresponding to pure components, therefore in such an estimation of molar heat capacity, molar entropy seems to be inaccurate for a mole fraction of acid more than 0.9, and less than 0.1, which can indicate that the assumption of the linear $H_{m}^{E}$ temperature shift becomes unreliable in extreme ranges. Consequently, estimation of all other thermodynamic properties remains reliable only within this concentration range.

The linear temperature dependence of the molar excess enthalpy leads to molar excess capacity remaining constant with the temperature shift which is a widespread approximation for a small temperature range. According to (15), the difference between $S_{m}^{E}$ for two temperatures may be calculated as:

(22) $$S_{T_{2},m}^{E}-S_{T_{1},m}^{E}=C_{p,m}^{E}\int_{T_{1}}^{T_{2}}\frac{dT}{T}=C_{p,m}^{E}\ln\frac{T_{2}}{T_{1}}$$

As the $\ln\frac{T_{2}}{T_{1}}$ is a constant for two defined temperatures, the shape of the $S_{T_{2},m}^{E}-S_{T_{1},m}^{E}$ dependence on composition will be similar to the $C_{p,m}^{E}-x$ curve.

We used literature data on vapor–liquid equilibria for the acetic acid + n-butanol system at 323.15 K [40] to calculate molar excess Gibbs energy at 323.15 K. We calculated the values of $G_{m}^{E}$ using the Raoult law assuming that the vapor of n-butanol is an ideal gas, that was proved to be a good approximation in some works [41]. The deviations of the acetic acid vapor from the ideal gas were taken into account by using associated ideal gas model. 

Then, we assumed that the discussed temperature range is small enough for extrapolating the linear dependence of $H_{m}^{E}$ on T. Thus, we could estimate the value of $H_{m}^{E}$ at 323.15 K. The $S_{m}^{E}$ at 323.15 K was calculated using Equation (17). Equation (22) was applied to calculate molar excess entropy at 313.15K. Since we have experimental $H_{m}^{E}$ values and estimated $S_{m}^{E}$ values at 313.15 K, Equation (16) allows estimating molar excess Gibbs energy at the same temperature. Molar excess Gibbs energy of a binary system relates to the molar Gibbs energy of mixing by the following equation:

(23) $$\Delta_{mix}G_{m}=G_{m}^{E}+RTx_{1}\ln x_{1}+RTx_{2}\ln x_{2}$$

**Figure 5:**
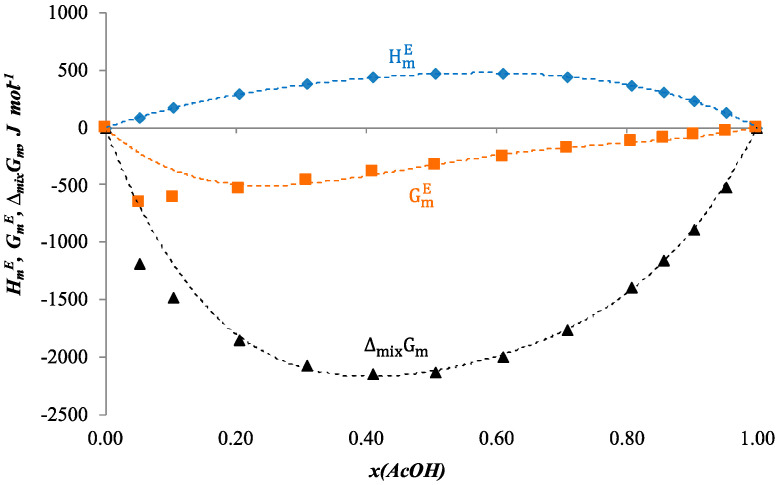
Molar excess enthalpy $H_{m}^{E}$, J mol^−1^ (♦) (our data), estimated molar excess Gibbs energy $G_{m}^{E}$, J mol^−1^ (■) and molar Gibbs energy of mixing $\Delta_{mix}G_{m}$, J mol^−1^ (▲) for the system acetic acid + n-butanol at 313.15K. The dotted lines (- - -) are the approximation polynomials, *x*—mole fraction of acetic acid.

Molar entropy of mixing was estimated by an equation similar to Equation (17). Calculated values of all the functions at 313.15 K are listed in Table 8. Dependence of estimated $S_{m}^{E}$ and $\Delta_{mix}S_{m}$ on composition of the solution is shown in Figure 4. Figure 5 represents the dependence of estimated $G_{m}^{E}$ and $\Delta_{mix}G_{m}$ on composition of the solution in comparison with the dependence of the $H_{m}^{E}$. The dotted lines are the approximation polynomials. 

For the estimation of uncertainties of calculated thermodynamic properties, we used the formula for uncertainty for a function of many variables:

(24) $$\Delta f\left(X_{1},X_{2},\ldots ,X_{n}\right)=\sqrt{{\left(\frac{\partial f}{\partial X_{1}}\Delta X_{1}\right)}^{2}+{\left(\frac{\partial f}{\partial X_{2}}\Delta X_{2}\right)}^{2}+\ldots +{\left(\frac{\partial f}{\partial X_{n}}\Delta X_{n}\right)}^{2}}$$

Using (17) and (20), we estimated the uncertainty of $S_{m}^{E}$ at 323.15 K as a function of $G_{m}^{E}$, $S_{m}^{E}$ and T. After that, we used calculated uncertainty of $S_{m}^{E}$ at 323.15K, calculated by Equation (21) of the uncertainty of $C_{p,m}^{E}$ and Equations (22) and (24) to estimate the uncertainty of calculated value of $S_{m}^{E}$ at 313.15K, which was 0.6 JK^−1^ mol^−1^. The values of molar excess entropy are even lower than molar excess heat capacity (average value = 2.0 JK^−1^ mol^−1^), which means that the relative standard uncertainty for calculated entropy will be even more (30%). A reasonable increase in the relative standard uncertainty is observed. Such an increase is caused by the accumulation of the uncertainty with further calculations. However, the main reason is the lack of experimental data presented in the literature.

Substituting (24) in (16), we evaluated the uncertainty of the calculated $G_{m}^{E}$, which was 100 J mol^−1^. Due to the low contribution of uncertainty of the composition, the uncertainty of the molar Gibbs energy of mixing could be assumed to be equal to the uncertainty of the molar excess Gibbs energy. According to the average values of Gibbs energy (−308 J mol^−1^ for $G_{m}^{E}$ and −1550 J mol^−1^ for $\Delta_{mix}G_{m}$), an increase in the relative standard uncertainty can be observed as well (32% $G_{m}^{E}$ and 6% for $\Delta_{mix}G_{m}$). Such a low uncertainty for the molar Gibbs energy of mixing is caused by a high accuracy of the composition according to (23) and (24). 

Although the relative uncertainties of the estimated functions are quite high, in the case of a limited data bank the estimation could be useful for qualitative assessments.

For the other two binary systems, the same estimation was not performed. For the system acetic acid + n-butyl acetate, only data at 313.15 K are available, so there is no possibility to calculate other properties. In the case of the n-butanol + n-butyl acetate binary system, literature data for different temperatures are rather sparse and the calculation seems to be unreliable. 

According to Figure 4 and Figure 5, it could be presumed that the estimations of the thermodynamic functions for the binary system acetic acid + n-butanol seem to be reliable at the concentration range 0.2 < x(AcOH) < 0.8.

## 4. Conclusions

According to the results of the study, new experimental data on molar excess enthalpy in binary subsystems of the system with the synthesis of n-butyl acetate were obtained. All experimental values were verified for consistency by the Redlich–Kister equation. The *ARD* for system acetic acid + n-butanol is 0.4%, for system acetic acid + n-butyl acetate it is 5% and for system n-butanol + n-butyl acetate it is 0.8%. The molar excess enthalpies data were also correlated with the NRTL model. The *ARD* between the experimental molar excess enthalpies and the predicted ones for acetic acid + n-butanol is 0.5%, for acetic acid + n-butyl acetate it is 24% and for the system n-butanol + n-butyl acetate it is 1.0%. In addition, a broad comparative analysis of the literature data was carried out, which showed that the results available in the literature on the molar heats of mixing for the acetic acid + n-butanol + n-butyl acetate + water system are not consistent with each other and require additional confirmation. According to our data and literature ones, some thermodynamic properties ($C_{p,m}^{E}$, $S_{m}^{E}$, $\Delta_{mix}S_{m}$, $G_{m}^{E}$ and $\Delta_{mix}G_{m}$) of the system acetic acid + n-butanol were estimated using equations of classical thermodynamics. The estimation seems to be reliable for quite a high concentration, however, for extremely high and low mole fractions of the acetic acid the error of the evaluation might be significant due to the lack of data that are necessary for the calculations. The knowledge of the presented excess thermodynamic properties is necessary in quantitative description of the deviations from ideality of the thermodynamic functions of solutions, which arise as a result of interaction between molecules through van der Waals forces, hydrogen bonds and others.

## Figures and Tables

**Table 1:** Literary data on molar excess enthalpy data for binary subsystems and quaternary system acetic acid + n-butanol + n-butyl acetate + water.

System	Temperature T/K	Comments	Literature
Acetic acid + n-butanol	298.15	Experimental data	[11]
	298.15, 318.15	Experimental data, graphs only	[12]
	313.15	Experimental data (NRTL)	[13]
Acetic acid + n-butyl acetate	313.15	Experimental data	[13]
Acetic acid + water	290.15, 293.15, 298.15, 303.15, 313.15, 323.15	Experimental data (fitted to the equation)	[14]
	293.15, 313.15	Calculated data (UNIQUAC model)	[15]
	293.15, 313.15	Calculated data (association model, +NRTL)	[16]
	293.15	Experimental data	[17]
	296.15–298.15	Experimental data (Redlich and Kister equation)	[18]
	298.15	Experimental data (fitted to the equation)	[19]
	298.15	Experimental data	[20]
	313.15	Experimental data (NRTL)	[13]
	313.15	Calculated (UNIFAC)	[21]
n-Butanol + n-butyl acetate	298.15	Experimental data (fitted to the equation)	[22]
	298.15	Experimental data (Redlich–Kister and SSF equations, UNIQUAC and NRTL models)	[23]
	298.15, 313.15	Experimental data (fitted to the equation)	[24]
	303.15	Experimental data (fitted to the equation)	[25]
	313.15	Experimental data (NRTL)	[13]
	353.15	Calculated data (Douglas–Avakian method)	[26]
n-Butanol + water	298.15	Experimental data (fitted to the equation)	[27]
	303.15	Experimental data	[28]
	303.15	Experimental data	[29]
	303.15, 328.15	Experimental data	[30]
	313.15	Experimental data (NRTL)	[13]
n-Butyl acetate + water	298.15	Experimental data (fitted to the equation)	[31]
	313.15	Experimental data (NRTL)	[13]
Acetic acid + n-butanol + n-butyl acetate + water	313.15	Experimental data (NRTL)	[13]

**Table 2:** Molar excess enthalpies of the acetic acid + n-butanol system at 313.15 K ^a^ (J mol^−1^), *x*—mole fraction of acetic acid.

*x*(AcOH)	$H_{m}^{E}$/J mol^−1^	*x*(AcOH)	$H_{m}^{E}$/J mol^−1^
0.0520	90.0	0.6092	475.2
0.1046	170.9	0.7082	443.8
0.2067	290.1	0.8063	369.4
0.3109	382.3	0.8563	307.7
0.4107	434.6	0.9032	229.3
0.5078	468.9	0.9521	128.0

^a^ Standard uncertainties of temperature *u*(*T*) = 0.05 K, mole fraction *u*(*x*) = 0.0001 and molar excess enthalpies is *U_r_*($H_{m}^{E}$) = 0.03 (95% level of confidence).

**Table 3:** Excess enthalpies of the acetic acid + n-butyl acetate system at 313.15 K ^a^ (J mol^−1^), *x*—mole fraction of acetic acid.

*x*(AcOH)	$H_{m}^{E}$/J mol^−1^	*x*(AcOH)	$H_{m}^{E}$/J mol^−1^
0.0505	39.2	0.5957	48.7
0.1011	76.6	0.7023	21.0
0.2014	100.2	0.7995	0.3
0.2994	100.7	0.8010	−0.6
0.4004	94.3	0.8992	−11.5
0.5006	70.6	0.9496	−11.2

^a^ Standard uncertainties of temperature *u*(*T*) = 0.05 K, mole fraction *u*(*x*) = 0.0001 and molar excess enthalpies is *U_r_*($H_{m}^{E}$) = 0.03 (95% level of confidence).

**Table 4:** Excess enthalpies of the n-butanol + n-butyl acetate system at 313.15 K ^a^ (J mol^−1^), *x*—mole fraction of acetic acid.

*x*(BuOH)	$H_{m}^{E}$/J mol^−1^	*x*(BuOH)	$H_{m}^{E}$/J mol^−1^
0.0527	376.9	0.5984	1465.8
0.0980	652.5	0.6982	1283.8
0.1972	1104.0	0.8044	962.5
0.2965	1392.9	0.9006	549.7
0.4006	1512.3	0.9499	291.1
0.4967	1566.3		

^a^ Standard uncertainties of temperature *u*(*T*) = 0.05 K, mole fraction *u*(*x*) = 0.0001 and molar excess enthalpies is *U_r_*($H_{m}^{E}$) = 0.03 (95% level of confidence).

**Table 5:** The purities of the chemicals.

CAS Number	Substance	Symbolic Name	Source	Purity, Mole Fraction	Purification Method	Analysis Technique
64-19-7	Acetic acid	AcOH	LenReactive (Russia)	0.998 ^b^	None	GC ^a^
71-36-3	n-Butanol	BuOH	Vekton (Russia)	0.995 ^b^	None	GC ^a^
123-86-4	n-Butyl acetate	BuOAc	Vekton (Russia)	0.999 ^b^	None	GC ^a^
7732-18-5	Water	H_2_O	Bidistill	0.999 ^b^	Distillation	GC ^a^

^a^ Gas chromatography. ^b^ Standard uncertainties of mole fraction *u*(*x*) = 0.005.

**Table 6:** Fitting parameters *A_k_*, for Equations (1)–(3) for binary mixtures of acetic acid + n-butanol, acetic acid + n-butyl acetate and n-butanol + n-butyl acetate with *ARD* and standard deviations, *σ*($H_{m}^{E}$, J mol^−1^).

Coeff.	AcOH (*i*) + BuOH (*j*)	AcOH (*i*) + BuOAc (*i*)	BuOH (*i*) + BuOAc (*i*)
A_0_	1867.1	287.6	6199.4
A_1_	−416.4	448.1	624.2
A_2_	547.9	60.1	952.0
A_3_	−160.0	213.5	239.9
A_4_		19.8	
*ARD* (%)	0.4	5	0.8
*σ*(*H^E^*)	1.1	1.3	6

**Table 7:** Binary interaction parameters of the NRTL model.

*i*	*j*	α*_ji_*	∆*g_ij_*	∆*g_ji_*	*ARD*/%
Acetic acid	n-Butanol	0.685	2425.3	1397.6	0.5
Acetic acid	n-Butyl acetate	0.024	−11,171.8	14,584.2	24
n-Butanol	n-Butyl acetate	0.213	4777.1	6170.1	1.0

**Table 8:** Estimated values of thermodynamic functions for the system acetic acid + n-butanol at 313.15 K ^a^.

x(AcOH)	$C_{p,m}^{E}$/JK^−1^ mol^−1^	$S_{m}^{E}$/JK^−1^ mol^−1^	$\Delta_{mix}S_{m}$/JK^−1^ mol^−1^	$G_{m}^{E}$/J mol^−1^	$\Delta_{mix}G_{m}$/J mol^−1^
0.0520	3.4	2.4	4.2	−6.5 × 10^2^	−11.8 × 10^2^
0.1046	3.4	2.5	5.3	−6.1 × 10^2^	−14.8 × 10^2^
0.2067	3.7	2.6	6.9	−5.3 × 10^2^	−18.6 × 10^2^
0.3109	4.4	2.7	7.9	−4.6 × 10^2^	−20.7 × 10^2^
0.4107	4.9	2.6	8.4	−3.9 × 10^2^	−21.5 × 10^2^
0.5078	5.2	2.5	8.5	−3.3 × 10^2^	−21.3 × 10^2^
0.6092	4.9	2.3	8.1	−2.6 × 10^2^	−20.0 × 10^2^
0.7082	4.2	2.0	7.2	−1.9 × 10^2^	−17.6 × 10^2^
0.8063	3.0	1.6	5.8	−1.2 × 10^2^	−14.0 × 10^2^
0.8563	2.2	1.3	4.8	−0.9 × 10^2^	−11.6 × 10^2^
0.9032	1.3	0.9	3.6	−0.6 × 10^2^	−8.8 × 10^2^
0.9521	0.4	0.5	2.1	−0.3 × 10^2^	−5.3 × 10^2^

^a^ Standard uncertainties of mole fraction *u*(*x*) = 0.0001, molar excess heat capacity is *u*($C_{p,m}^{E}$) = 0.5 JK^−1^ mol^−1^, molar excess entropy is *u*($S_{m}^{E}$) = 0.6 JK^−1^ mol^−1^, molar entropy of mixing is *u*($\Delta_{mix}S_{m}$) = 0.6 JK^−1^ mol^−1^, molar excess Gibbs energy is *u*($G_{m}^{E}$) = 1.0 × 10^2^ J mol^−1^, molar Gibbs energy of mixing is *u*($\Delta_{mix}G_{m}$) = 1.0 × 10^2^ J mol^−1^ (95 % level of confidence).

## Data Availability

Not applicable.
